# Diphtheria—A Circular from Dr. Norton

**Published:** 1865-10

**Authors:** 


### Diphtheria—A Circular from Dr. Norton.

Dr. O. D. Norton of Cincinnati, was placed on a special committee by the American Medical Association to report on Diphtheria. He has handed us the following list of queries, and any of our readers who have had opportunities for observing the disease, will confer a favor that will be appreciated and acknowledged, by corresponding with Dr. Norton:

I. Has Diphtheria occurred in your practice? If so, when did it first make its appearance? (Please state the year and the months in which it prevailed, and how many cases came under your observation.)

II. Did it occur as a Sporadic, Endemic or Epidemic disease?

III. Did the disease affect one class or age more particularly, and what were its general characteristics?

IV. What in your opinion are the general and what the exciting cause or causes of the disease?

V. Do you consider Diphtheria and Scarlatina identical?

VI. Do you consider it communicable?

VII. What other diseases were especially prevalent at the time?

VIII. Do you know of any disease having affected animals during the occurrence of Diphtheria in the community?

IX. Have you seen the Diphtheritic membrane developed upon the cutaneous surface or upon wounds?

X. In what proportion of cases has the disease invaded the Larynx? Also, the Œsophagus?

XI. What have been the Sequelæ?

XII. What has been the result of Post Mortem or Microscopical Examinations?

XIII. In what proportion of cases have you found Albumen in the Urine?

XIV. What has been the rate of mortality? and what the immediate cause of death?

XV. What was the general course of treatment pursued by you, and what particular remedial agents seemed to be most productive of good?—*Cincinnati Lancet.*

